# SR and LR Union Suture for the Treatment of Myopic Strabismus Fixus: Is Scleral Fixation Necessary?

**DOI:** 10.1155/2015/470473

**Published:** 2015-04-12

**Authors:** Carol P. S. Lam, Jason C. S. Yam, Flora H. S. Lau, Dorothy S. P. Fan, C. Y. Wong, Christopher B. O. Yu, Winnie W. Y. Lau

**Affiliations:** ^1^Department of Ophthalmology and Visual Sciences, The Chinese University of Hong Kong, Hong Kong Eye Hospital, Kowloon, Hong Kong; ^2^Department of Ophthalmology, Hong Kong Sanitorium and Hospital, Happy Valley, Hong Kong

## Abstract

*Purpose*. To evaluate and compare the effectiveness of scleral fixation SR and LR union suture and nonscleral fixation union suture for the treatment of myopic strabismus fixus. *Methods*. Retrospective review of 32 eyes of 22 patients with myopic strabismus fixus who had undergone union suture of superior rectus (SR) and lateral rectus (LR) with or without scleral fixation, and follow-up longer than 6 months at Hong Kong Eye Hospital from 2006 to 2013. Surgical techniques and outcomes in terms of ocular alignment are analyzed. *Results*. There is significant overall improvement both in postoperative angle of esodeviation (*P* < 0.01) and postoperative range of movement (*P* = 0.042). Comparing between the sclera fixation group (11 eyes) versus nonscleral fixation group (21 eyes), the postoperative horizontal deviation, the postoperative vertical deviation, successful outcome, and the change in horizontal deviation were not significantly different (*P* > 0.05). *Conclusions*. Union suture of SR and LR is an effective procedure in correcting myopic strabismus fixus. Fixation of the union suture to the sclera does not improve surgical outcome.

## 1. Introduction

Myopia is a prevalent disease in Hong Kong. Edwards and Lam showed more than 70% of Hong Kong adults were myopic [1]. Moreover, patients with pathological myopia had a high prevalence of horizontal and vertical strabismus [2]. Therefore, strabismus related to pathological myopia is not an uncommon problem in our locality. Myopic strabismus fixus, also known as acquired progressive esotropia associated with severe myopia (APEASM), or heavy eye syndrome, is an adult onset esotropia associated with high axial myopia, typically described as progressive esotropia and hypotropia associated with restricted elevation and abduction of variable degree and severity. Studies had shown that superotemporal herniation of the elongated eyeball beyond the muscle cone caused by the pathogenesis of myopic strabismus fixus [3–5]. Normally, the orbital connective tissue acts like a pulley supporting the extraocular muscles. The elongated eyeball mechanically compresses into the pulley, which leads to failure to maintain the normal position of extraocular muscles and prolapse of the elongated posterior eyeball. Therefore, the lateral rectus (LR) muscle is displaced inferiorly and superior rectus (SR) muscle is displaced nasally, resulting in limitation or failure to abduct and elevate. As our patients often present late, it often results in significant cosmetic concerns and technical difficulties for ophthalmological examinations.

Many surgical methods had been described so far, including vertical muscle transposition, ipsilateral medial rectus recession and ipsilateral lateral rectus resection, and posterior fixation suture [6]. However, treatment is difficult and recurrence of large angle esotropia and/or hypotropia after surgery is common. In 2001, loop myopexy technique was described by Yokoyama et al. [7] by joining the muscle bellies of SR and LR results in a sling of muscle that supports the elongated eye, pushing it back into the muscle cone. Several reports have also supported the effectiveness of this surgery [8–12]. Wong et al. reported two cases, one treated with traditional scleral fixation and the other with silicon band without scleral fixation [8]. Leo and del Monte reported a single case report in which the myopexy was performed together with medial and inferior rectus recession and 3-point scleral fixation [11]. Durnian et al. reported five cases of myopexy of the superior and lateral rectus without scleral fixation or medial rectus recession [12]. There were two relatively large series reported recently. Fresina et al. reported a series of 33 eyes of 26 patients underwent muscle belly union without scleral fixation [13]. Shenoy et al. reported a series of 26 eyes of 15 patients with silicone band loop myopexy of LR and SR with the use of a scleral tunnel [14].

As scleral fixation on pathological myopic eyes could impose potential risk of scleral perforation, our study aims to report the surgical techniques and evaluates and compares the results of patients with myopic strabismus fixus patients who had undergone union suture of SR and LR with or without scleral fixation.

## 2. Method

This is a retrospective case series of thirty-two eyes of twenty-two patients who had undergone union suture of LR and SR for myopic strabismus fixus at Hong Kong Eye Hospital from 2006 to 2013. This study was approved by ethic committee in the KC/KE cluster of Hospital Authority in Hong Kong and the study was carried out in accordance with the approved guidelines. Informed consent was obtained from all subjects before surgery.

**Figure 1:**
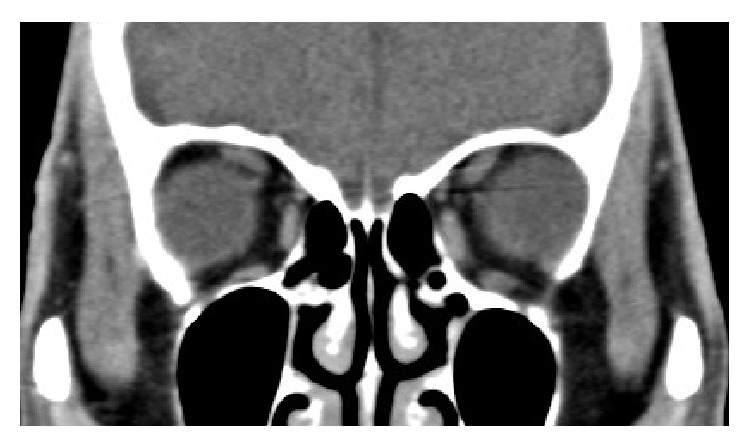
Coronal cut of computed tomography of the orbit of a patient showing superotemporal herniation of the eyeball with inferior displaced LR and nasally displaced SR muscles.

In our cohort, all patients were diagnosed to have myopic strabismus fixus preoperatively, as confirmed by computed tomography of orbit and brain, showing superotemporal herniation of the elongated eyeball from the muscle cone, inferior displacement of the LR, and nasal displacement of the SR. Other intracranial related pathologies are excluded (Figure 1). Patients with less than 6 months follow-up were excluded.

The degree of esotropia and hypotropia was assessed preoperatively and postoperatively for each patient by prism bar cover test (PBCT). In the situation that PBCT could not be performed due to extreme limitation of extraocular muscle movement, the degree of ocular deviation was estimated by the Krimsky test [15, 16].

The degree of restriction of extraocular movement was graded into 4 grades of impairment. Grade 1 is small restriction; Grade 2 is moderate restriction; Grade 3 is severe restriction in which the eye could not cross midline; Grade 4 is extreme restriction with the eye fixed in adduction with minimal movement. The degrees of restriction are reported preoperatively and postoperatively.

Individual age, sex, duration of squint, visual acuity, refractive status, axial length, associated ocular disease, any previous squint operations, and associated complications are all reported.

Successful outcome was defined as residual esotropia less than 10 PD or consecutive exotropia less than 10 PD.

### 2.1. Surgical Techniques

All patients had surgery under general anesthesia. Forced duction tests were performed to confirm the restriction. Union suture of SR and LR was performed for all patients as described below.

**Figure 2:**
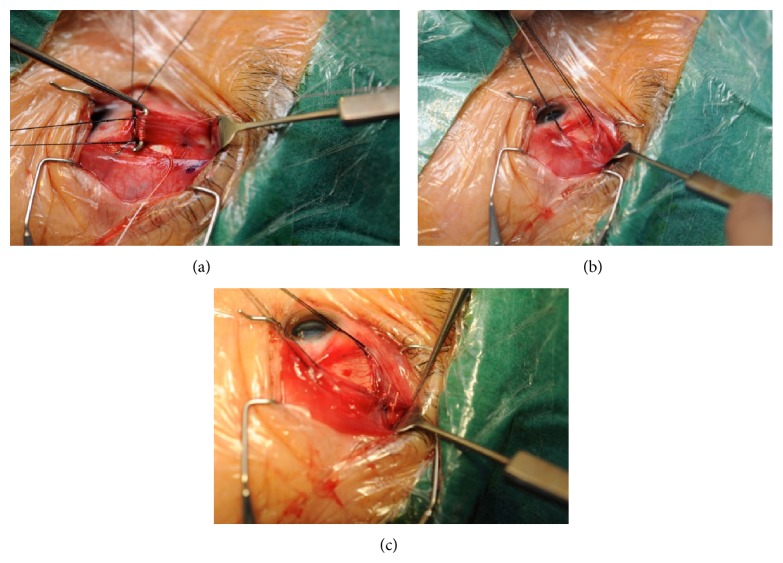
(a) The LR and SR muscles were identified through a forniceal conjunctival incision in the superotemporal quadrant and traction sutures were placed onto the LR and SR muscles. (b) The first union suture joining the superior half of the LR and temporal half of the SR at 10 mm after muscle insertion using 5-O nonabsorbable polyester suture; (c) the second union suture is made similarly at 15 mm after muscle insertion using 5-O nonabsorbable polyester suture.

A forniceal conjunctival incision is created in the superotemporal quadrant approximately 8 mm posterior to the limbus. LR and SR muscles are then identified and isolated with a 4'O silk suture (Mersilk, Ethicon). The temporal half of the SR muscle and the superior half of the LR muscle are sutured with a nonabsorbable 5-O polyester suture and then united together at 2 sites (10 mm and 15 mm post muscle insertion), with or without a scleral fixation suture (Figure 2) depending on surgeon preference. The SR and LR muscles were not disinserted.

If the union suture alone was insufficient to recover the movable range of the globe due to very tight medial rectus (MR) and inferior rectus (IR), concurrent ipsilateral recession or disinsertion of MR and/or IR recession may be performed. The MR and IR muscles were sutured with 6'O vicryl (coated vicryl, Ethicon) by hang-back technique.

### 2.2. Statistical Analysis

To evaluate the surgical efficacy of the procedure, we compared the angle of deviation and degree of extraocular movement as stated above preoperative and postoperatively to analyze the effectiveness of SR/LR union suture with or without scleral fixation.

Subgroup analysis was calculated by Chi-square test, Mann-Whitney *U* test and Kolmogorov-Smirnov test with the aid of software SPSS (version 16.0, SPSS Inc., Chicago, Illinois, USA). All tests were considered to be statistically significant if *P* value was <0.05.

## 3. Results

32 eyes of 22 patients were recruited in the cohort. 14 were female and 8 were male. Age ranged from 37 to 87 year old (median = 64 years old). Postoperative follow-up period ranged from 6 months to 75 months (mean = 30 months).

### 3.1. Characteristics of the Operated Eyes

Best Snellen corrected visual acuity of the operated eyes ranged from light perception to 0.4 (median = 0.1). Axial length of the operated eyes ranged from 27 mm to 36.77 mm (mean = 30.80). The spherical equivalent of the refractive error of the operated eyes ranged from −9.00 D to −26.00 D (mean = 19.08). Among the 32 operated eyes, 12 had concurrent cataract; 12 were pseudophakic; 4 had glaucoma; 15 had myopic maculopathy.

### 3.2. Union Sutures Procedures Performed

Out of 22 patients, two had bilateral eyes procedures done concurrently; eight patients had consecutive procedures done in each eye; 12 patients received single eye surgery. In total, 30 union sutures procedures had been performed in 32 eyes of 22 patients. Scleral fixation sutures were performed in 11 eyes and nonscleral fixation sutures in 21 eyes.

### 3.3. Prior Strabismus Operations

Among the 22 patients, 10 had received prior strabismus operations. 3 had ipsilateral MR recession and LR resection was done on the operated eyes; 2 had MR recession and LR resection done for the fellow eyes; 1 had both eyes MR recession and LR resection done; 1 had ipsilateral IR recession and MR recession done.

### 3.4. Concurrent Strabismus Operations

Among the 32 eyes with union suture done, 21 had concurrent ipsilateral MR recession done; 4 had concurrent ipsilateral MR disinsertion done; 4 had ipsilateral IR recession done. The amount of MR recession performed ranged from 4 mm to 11.5 mm (median = 7.5 mm). The amount of IR recession performed ranged from 5 mm to 7 mm (median 6.5 mm). 11 eyes had the first union suture of LR and SR together with scleral fixation done.

### 3.5. Surgical Outcomes

Preoperatively, the mean angle of esodeviation was 80.9 ± 24.25 PD. The mean angle of deviation was 6.93 ± 27.37 PD; 7.33 ± 28.30 PD; and 7.35 ± 30.66 PD at postoperative 1 week, 3 months, and 1 year, respectively. Out of all union procedures, 20 (66.7%), 19 (63.3%), and 19 (73.1%) had successful outcome at 1 week (*n* = 30), 3 months (*n* = 30), and 1 year (*n* = 26), respectively. There is significant improvement in postoperative angle of esodeviation at 1 week, 3 months, and 1 year follow-up compared with the preoperative angle (*P* < 0.01, Kolmogorov-Smirnov test). All union procedures except two resulted in improvement in esodeviation. There was one case with no change of ocular deviation and one case with consecutive exotropia of 95 PD.

In regards for the vertical deviation, the mean angle of hypotropia was 24.00 ± 15.57 PD (*n* = 5) and 2.36 ± 8.64 PD (*n* = 14) preoperatively and at postoperation 3 months, respectively. The number of cases with vertical deviation reported was too small for meaningful statistical analysis.

The degree in range of extraocular movement improvement was measured from 3 months to 6 months postoperatively. Two eyes had no improvement in movement; 9 eyes had 1 grade of improvement; 13 eyes had 2 grades of improvement; 6 eyes had 3 grades of improvement; 2 eyes had 4 grades of improvement. There was significant improvement in range of movement after surgery (*P* = 0.042, Kolmogorov-Smirnov test).

### 3.6. Scleral Fixation Group versus Nonscleral Fixation Group

Baseline characteristics and clinical outcome parameters between scleral fixation and nonscleral fixation group were summarized in Table 1. The baseline characteristics are similar between two groups (*P* > 0.05). The postoperative horizontal deviation, the postoperative vertical deviation, and the change in horizontal deviation were not significant different between the two groups (*P* > 0.05).

Among those with scleral fixation, 70.0% at 1 week, 70.0% at 3 months, and 71.4% at 1 year had successful outcome. Among those without scleral fixation, 65.0% at 1 week, 60.0% at 3 months, and 73.7% at 1 year had successful outcome.

## 4. Discussion

Union suture of SR and LR is an effective treatment for patients with myopic strabismus fixus as it can reduce the degree of esotropia and increase in range of extraocular movement by a significant amount. As proposed by Yamaguchi et al. [5], the surgery helps to normalize the vectors of muscle force of the SR and LR, allowing the globe to move more freely within the muscle cone by eliminating the mechanical disturbance of eye movement.

As compared with the method of Yamada et al. proposed hemitransposition of the SR and LR [17], we preferred union suture of SR and LR because no muscle detachment of SR and LR was involved. Therefore, this would potentially decrease the risk of anterior segment ischemia and provide a safer and faster operation.

We found no significant difference in the scleral fixation group and nonscleral fixation group in our case series. As scleral fixation on pathological myopic eyes could impose potential risk of scleral perforation, this might not be a necessary procedure as most of our cases with no scleral fixation done still obtained satisfactory surgical outcome.

It was noted that the sample size of the reported vertical deviation was reduced, which is more significant before surgery. This is due to the difficulty in measuring the vertical deviation when the horizontal deviation was too large. Most of our cases had large angle esotropia (mean 80.9 PD ± SD 24.25 PD) with their eyes kept in fixed adducted position before surgery.

The reason for the MR disinsertion in two of our cases was due to extreme fibrosis and the muscle was broken during MR recession procedure. Surgical outcome of muscle disinsertion could be unpredictable at times as one of our patients was noted to have significant exotropia larger than 95 PD after surgery. It is observed that all cases with controlled MR recession had no reported cases of consecutive divergent squint. Therefore, we proposed that early diagnosis and surgery is advised so as to avoid surgery on fibrotic MR/IR and also to prevent the complication of having a disinserted muscle.

Our study is not without limitation. This is a retrospective study with relatively small sample size and nonrandomized treatment given to the cases. However, because of the rarity of this disease, our case series is one of the largest series reported.

## 5. Conclusion

Union suture of SR and LR is an effective procedure in treating patients suffering from myopic strabismus fixus. Fixation of the union suture to the sclera does not improve surgical outcome.

## Figures and Tables

**Table 1:** Baseline characteristics and postoperative clinical outcomes between scleral fixation and nonscleral fixation groups.

	Scleral fixation group	Nonscleral fixation group	*P* value
Baseline characteristics			

Sex (M : F) (*N* = 30)	1 : 1	1 : 3	0.17^a^
Age (year) (*N* = 30)	65.00 ± SD 16.44	68.84 ± SD 8.19	0.88^b^
Previous squint operations (*N* = 30) (None : previous procedure)	1 : 1	4 : 1	0.11^a^
Axial length (mm) (*N* = 23)	30.40 ± SD 2.08	31.71 ± SD 2.05	0.23^b^
Concurrent MR surgery (absent/present) (*N* = 30)	1 : 9	1 : 3	0.33^a^

Follow-up (year) (*N* = 30)	2.00 ± SD 1.45	3.63 ± SD 2.32	0.19^b^
Preoperative horizontal deviation (PD) (*N* = 30)	72.5 ± SD 26.90	85.15 ± SD 22.34	0.19^b^
Preoperative vertical deviation (PD) (*N* = 5)	17.50 ± SD 10.61	28.33 ± SD 18.93	0.56^b^

Postoperative clinical outcomes			

Postoperative horizontal deviation			
1 week (PD) (*N* = 30)	9.42 ± SD 14.22	5.37 ± SD 33.33	0.71^b^
3 months (PD) (*N* = 30)	10.00 ± SD 17.32	5.79 ± SD 34.07	0.96^b^
12 months (PD) (*N* = 26)	13.57 ± SD 20.15	5.05 ± SD 33.91	0.39^b^

Postoperative vertical deviation			
3 months (PD) (*N* = 14)	−1.25 ± SD 13.00	3.80 ± SD 6.58	0.88^b^

Change in horizontal ocular deviation			
1 week (PD) (*N* = 30)	59.86 ± SD 21.99	81.63 ± SD 39.60	0.29^b^
3 months (PD) (*N* = 30)	59.29 ± SD 20.90	81.21 ± SD 39.36	0.27^b^
12 months (PD) (*N* = 26)	55.71 ± SD 21.88	81.95 ± SD 38.97	0.06^b^

% of surgical success			
1 week (%) (*N* = 30)	70.0	65.0	0.78^a^
3 months (%) (*N* = 30)	70.0	60.0	0.59^a^
12 months (%) (*N* = 26)	71.4	73.7	0.91^a^

^a^Chi-square test; ^b^Mann-Whitney *U* test.
